# Piperine enhances doxorubicin sensitivity in triple-negative breast cancer by targeting the PI3K/Akt/mTOR pathway and cancer stem cells

**DOI:** 10.1038/s41598-024-65508-0

**Published:** 2024-08-06

**Authors:** Andrew N. Hakeem, Dina M. El-Kersh, Olfat Hammam, Aliaa Elhosseiny, Amr Zaki, Kohinour Kamel, Lidia Yasser, Marina Barsom, Menatallah Ahmed, Mohamed Gamal, Yasmeen M. Attia

**Affiliations:** 1 Pharmacology Department, Faculty of Pharmacy, The British University in Egypt, Cairo, Egypt; 2 Health Research Center of Excellence, Drug Research and Development Group, Faculty of Pharmacy, The British University in Egypt, Cairo, Egypt; 3 Pharmacognosy Department, Faculty of Pharmacy, The British University in Egypt, Cairo, Egypt; 4 Pathology Department, Theodor Bilharz Research Institute, Giza, Egypt; 5 Graduate Students, Faculty of Pharmacy, The British University in Egypt, Cairo, Egypt; 6 Biochemistry Department, Faculty of Pharmacy, The British University in Egypt, Cairo, Egypt

**Keywords:** Triple-negative breast cancer, Piperine, Doxorubicin, Cancer stem cells, PI3K/Akt/mTOR, PTEN, Cancer, Chemical biology, Diseases, Oncology

## Abstract

Triple-negative breast cancer (TNBC) is an aggressive subtype of breast cancer that lacks an actionable target with limited treatment options beyond conventional chemotherapy. Therapeutic failure is often encountered due to inherent or acquired resistance to chemotherapy. Previous studies implicated PI3K/Akt/mTOR signaling pathway in cancer stem cells (CSCs) enrichment and hence chemoresistance. The present study aimed at investigating the potential effect of piperine (PIP), an amide alkaloid isolated from *Piper nigrum*, on enhancing the sensitivity of TNBC cells to doxorubicin (DOX) in vitro on MDA-MB-231 cell line and in vivo in an animal model of Ehrlich ascites carcinoma solid tumor. Results showed a synergistic interaction between DOX and PIP on MDA-MB-231 cells. In addition, the combination elicited enhanced suppression of PI3K/Akt/mTOR signaling that paralleled an upregulation in this pathway’s negative regulator, PTEN, along with a curtailment in the levels of the CSCs surrogate marker, aldehyde dehydrogenase-1 (ALDH-1). Meanwhile, in vivo investigations demonstrated the potential of the combination regimen to enhance necrosis while downregulating PTEN and curbing PI3K levels as well as p-Akt, mTOR, and ALDH-1 immunoreactivities. Notably, the combination failed to change cleaved poly-ADP ribose polymerase levels suggesting a pro-necrotic rather than pro-apoptotic mechanism. Overall, these findings suggest a potential role of PIP in decreasing the resistance to DOX in vitro and in vivo, likely by interfering with the PI3K/Akt/mTOR pathway and CSCs.

## Introduction

Breast cancer is the most prevalent malignancy and the leading cause of cancer-related mortality in women globally^1^. Triple-negative breast cancer (TNBC) is a heterogeneous disease representing one poorly characterized subtype of breast cancer that, by definition, does not meet the diagnostic threshold for the expression of the hormone receptors (estrogen/progesterone) or the human epidermal growth factor receptor (HER)-2^2^. TNBC afflicts 15–20% of breast cancer patients and compared to other breast malignancies, it has the worst prognosis in terms of tumor relapse and patient survival^3^. This makes it a clinically aggressive type of cancer with no targeted therapy available. Non-specific systemic chemotherapy, including anthracycline/platinum/taxane-based regimens, thus remains the primary therapeutic option for TNBC patients alongside radiotherapy and tumor resection tailored on a case-by-case basis^4^. However, TNBC patients encounter two major limitations, adverse events associated with the use of a high-dose regimen and therapeutic failure due to intrinsic or de novo resistance^5^. This provides a rationale for adopting combinatorial therapies in TNBC where preventing dose-escalation and decreasing the chances of chemoresistance comprise the most prominent treatment goals^6^.

Doxorubicin (DOX), an anthracycline inhibitor of DNA topoisomerase II, lies chief among chemotherapeutics utilized in TNBC. Nevertheless, DOX-induced cardiotoxicity precludes its use in TNBC patients with low ejection fraction and is the reason why these patients are screened for cardiac functions before even being considered eligible for an anthracycline-based regimen^7^. On the other hand, despite the attempts of deciphering the mechanisms underlying DOX chemoresistance, this remains an unresolved issue.

The PI3K/Akt/mTOR signaling pathway has increasingly been linked to cancer stem cells (CSCs) in a variety of solid tumors, including TNBC, thus conferring chemoresistance and tumor relapse. Moreover, gain-of-function mutations in PI3K/Akt/mTOR signaling pathway and loss-of-function mutations in its negative regulator, the phosphatase and tensin homolog (PTEN), are frequently observed in TNBC, accounting for approximately 25% of primary TNBC tumors, and are hypothesized to be even more frequent in metastatic TNBC^8^. In fact, aberrations in PI3K/Akt/mTOR are the second most frequent after that of TP53 in patients with TNBC^9^. Such observation spurred clinical interest towards leveraging PI3K/Akt/mTOR targeted therapies which is evident by a number of inhibitors undergoing phase-II/III clinical trials in TNBC^8,9^. Intriguingly, PI3K signaling was also linked to DOX resistance in TNBC through regulating epithelial-to-mesenchymal transition (EMT) and hence the pool of CSCs^10,11^.

Piperine (PIP) is an amide alkaloid found in the fruits of black pepper, *Piper nigrum*, and has attracted attention for its anticancer effects across various malignancies. In colorectal cancer, it has been shown to arrest cell cycle progression and trigger apoptosis by modulating critical molecular pathways like the Wnt/β-catenin pathway^12^. Previous studies also reveal PIP’s potential to hinder metastasis in lung cancer through impeding EMT^13^. Its ability to enhance the efficacy of chemotherapeutic drugs also presents a compelling case for its use in combination-based regimens^14^. Moreover, PIP’s potential to inhibit proinflammatory cues provoked by nuclear factor-kappa B alongside others in melanoma was also reported^15^. Beyond its pro-apoptotic, anti-proliferative, and anti-metastatic capacities, the versatility of PIP-mediated actions extends to include effects that address chemoresistance, particularly in breast cancer^16^. Specifically, long-term treatment of DOX-resistant MCF-7 cells with PIP resulted in a significant decrease in ATP-binding cassette transporter-1 expression thereby downregulating the efflux transporter P-glycoprotein-1^17^, an effect that was subsequently substantiated in other cell lines and pre-clinical models^18^. This study, therefore, aimed at probing the potential impact of PIP on DOX cytotoxicity in TNBC and chemoresistance in vitro, emphasizing on the role of PI3K/Akt/mTOR signaling and its association with CSCs. An Ehrlich ascites carcinoma (EAC) solid tumor animal model was also used to validate the in vitro findings within the tumor microenvironment reflecting on the combination's capacity to induce apoptosis, necrosis, and expression of CSC markers.

## Results

### Identification of isolated PIP by 1D-NMR (^1^H & ^13^C)

**Figure 1:**
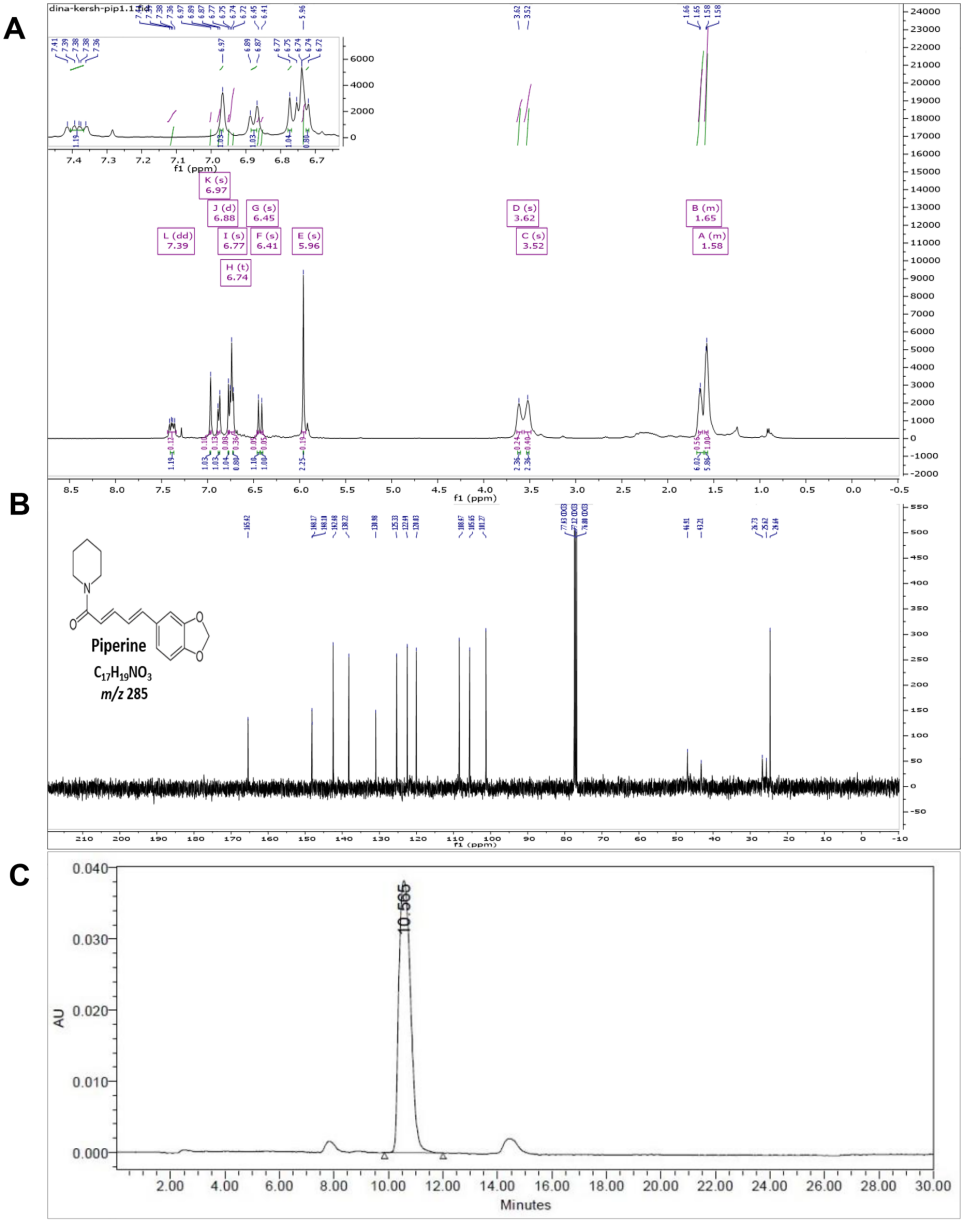
Purity and identity of piperine. 1D-NMR of piperine isolated from *P. nigrum* ethanolic extract. (**A**) ^1^H NMR and (**B**) ^13^C NMR. (**C**) The chromatographic HPLC–PDA run of piperine at 345 nm.

Identity of isolated PIP (C17H19NO3) was confirmed by 1D-NMR spectroscopy. In the ^1^H NMR spectrum (Fig. 1A), significant peaks of PIP have been identified. Signals at δ: 1.58–1.65 (6H, m), 3.52 (2H, s) and 3.62 (2H, s). A signal at δ 5.96 (2H, *s*) of methylene dioxy proton. Two broad (br) peaks at δ 6.41 (1H, br s) and δ 6.45 (1H, br s). A signal at δ 6.74 (1H, br s) and another one at δ 6.77 (1H, br s) appears as broad signal. Another signal at δ 6.87 (1H, br *d*) appears as broad doublet. A peak at δ 6.97 (1H, br s) appears as broad signal of the aromatic proton. Peak at δ 7.38 (1H, *dd*). Peaks that were detected at δ 6.74 and 6.77 signify the presence of PIP structure not isopiperine. All ^1^H-NMR peaks agreed with that reported previously^19^. In the ^13^C NMR spectrum (Fig. 1B), signals at 24.6, 25.6, 43.2, 46.9, 101.27, 105.65, 108.47, 120.03, 122.49, 125.33, 130.98, 138.22, 142.48, 148.10, 148.17 ppm were identical for PIP structure. Another signal characteristic for carbonyl group appeared at 165.4 ppm. All significant peaks were in accordance with previous data^19,20^.

### Purity of isolated PIP via HPLC–PDA

The purity of isolated PIP was confirmed via HPLC–PDA. The PIP major peak was observed in Fig. 1C at retention time (Rt.) 10.565 min at 345 nm which declared the purity of the isolated PIP.

### PIP enhances the sensitivity of MDA-MB-231 cells to DOX

**Figure 2:**
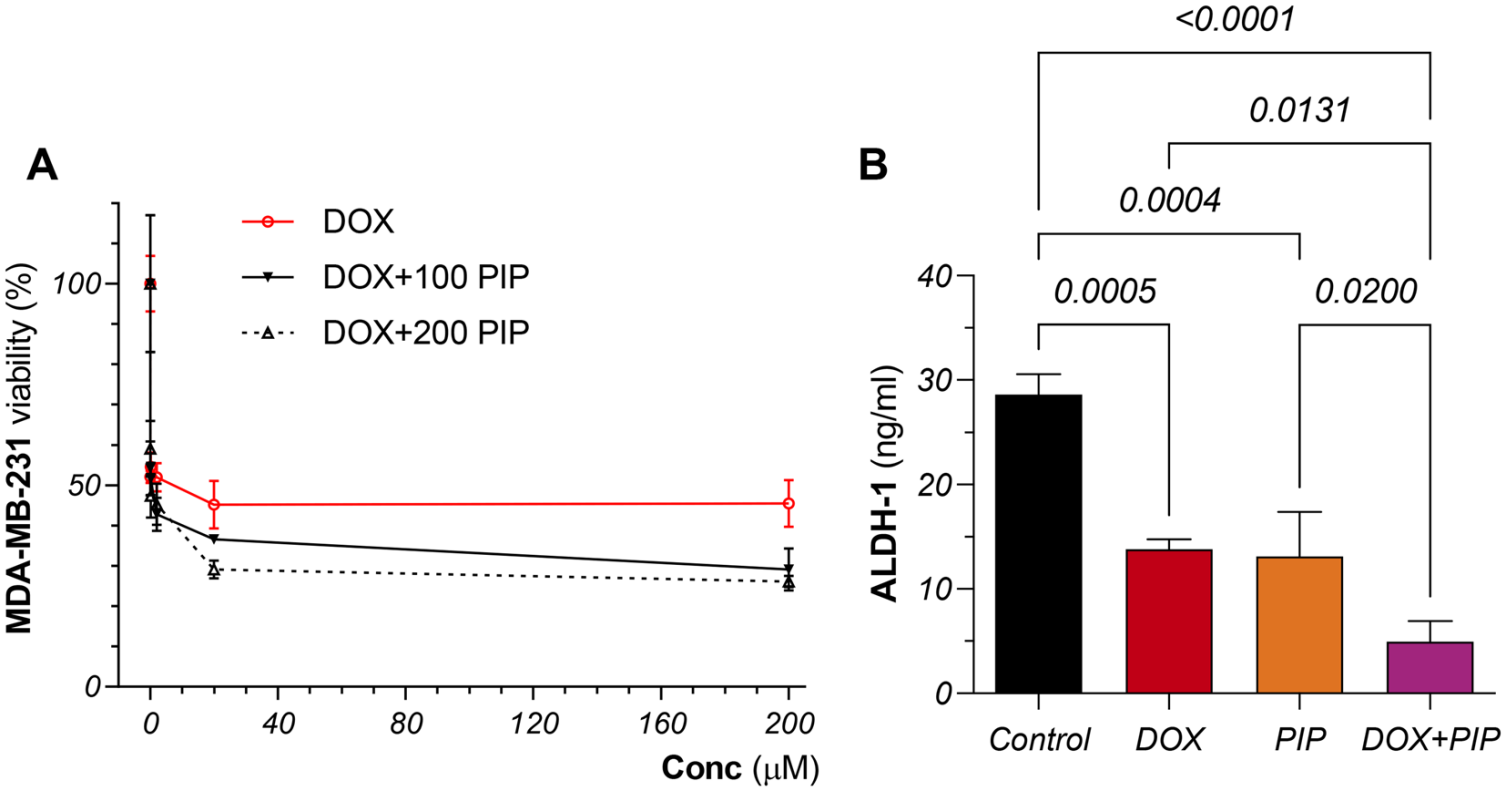
Cytotoxicity studies and effect of PIP on ALDH-1 protein levels in DOX-treated MDA-MB-231 cells. (**A**) Dose–response curve showing the effect of DOX alone and combined with PIP (100 and 200 µM) on the viability of MDA-MB-231 cells. (**B**) ALDH-1 protein levels in MDA-MB-231 cell lysates exposed to DOX and PIP, each alone and combined. Viability was detected using MTT assay. ALDH-1 levels were estimated using ELISA. Statistical difference was tested using one-way ANOVA, followed by Tukey’s multiple comparison test and significance was inferred for *P* < 0.05. Results are presented as means ± S.D. PIP, piperine; DOX, doxorubicin; ALDH-1, aldehyde dehydrogenase-1.

Cytotoxicity assays were carried out to attest if, and to what extent, culturing cells with PIP would impact the sensitivity of the TNBC cell line, MDA-MB-231, towards DOX treatment. As shown in Fig. 2A, treatment with DOX had an estimated IC_50_ value of 1.85 µM and confidence limit (CL) of − 0.15 to 23.52 whereas treatment with PIP elicited an IC_50_ of 415.2 µM (CL: 236.4–1809). Furthermore, combining PIP at 100 and 200 µM concentrations to DOX resulted in 86 and 90% higher cytotoxic effects than DOX alone. It is noteworthy that the combination indices (CI) for both concentrations of PIP were found to exhibit synergy with DOX (Table 1).

**Table 1:** The IC_50_ values of DOX and PIP, each alone and combined, along with their combination indices (C.I.) on MDA-MB-231 cells.

Treatment(s)	IC_50_ (µM)	C.I.
DOX	1.85	–
PIP	415.2	–
DOX + 100 µM PIP	0.16	0.33 (synergistic)
DOX + 200 µM PIP	0.19	0.59 (synergistic)

C.I., combination index; DOX, doxorubicin; IC_50_, half maximal inhibitory concentration; PIP, piperine.

### PIP enhances DOX-induced suppression of ALDH-1 in MDA-MB-231 cells

As a CSCs surrogate marker, ALDH-1 protein levels were estimated to investigate the potential effect of DOX + PIP treatment on CSCs. As shown in Fig. 2B, DOX and PIP single treatment caused a reduction in ALDH-1 levels reaching 52% and 55% compared to control untreated cells, respectively. On the other hand, DOX + PIP combination was superior to single treatments in curbing ALDH-1 levels, where the reduction in this group reached 60%, as compared to DOX alone.

### PIP enhances DOX-induced suppression of PI3K/Akt/mTOR signaling in MDA-MB-231 cells and upregulation in PTEN

**Figure 3:**
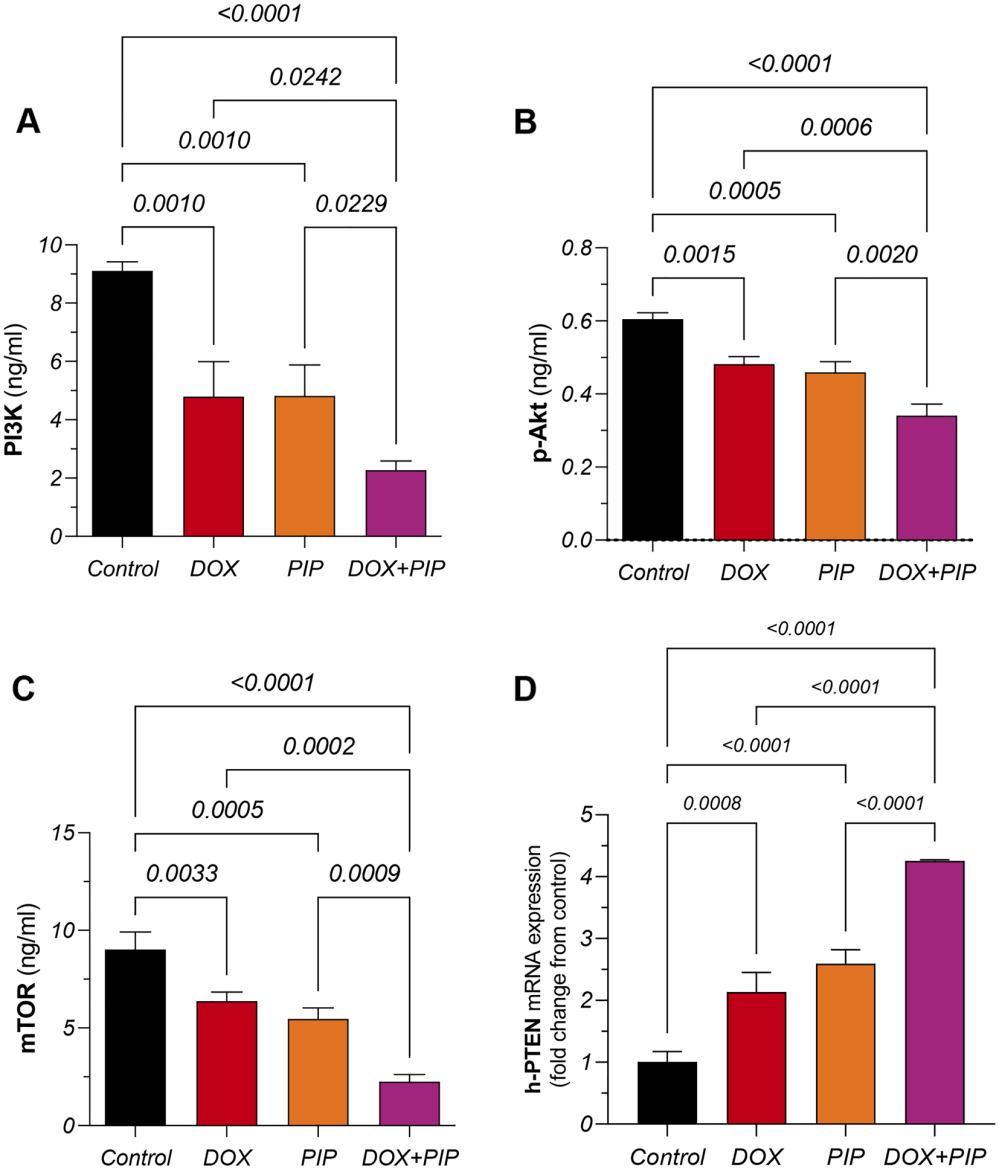
Effect of combining PIP to DOX on PI3K/p-Akt/mTOR/PTEN in MDA-MB-231 cells. Protein levels of (**A**) PI3K, (**B**) p-Akt, and (**C**) mTOR as well as the gene expression of (**D**) PTEN in MDA-MB-231 cell lysates exposed to DOX and PIP, each alone and combined. PI3K, p-Akt, and mTOR levels were estimated using ELISA. PTEN relative gene expression is expressed as fold change from control and was determined by qRT-PCR using delta-delta Ct (ΔΔCt) following normalization to the housekeeping β-actin gene. (**D**) Statistical difference was tested using one-way ANOVA, followed by Tukey’s multiple comparison test and significance was inferred for *P* < 0.05. Results are presented as means ± S.D. PIP, piperine; DOX, doxorubicin; PI3K, phosphatidyl inositol 3-kinase; mTOR, mammalian target of rapamycin; PTEN, phosphatase and tensin homolog.

Since PI3K/Akt/mTOR was previously linked to ALDH-1 expression in cancer, signal cascade members were estimated in MDA-MB-231 cells exposed to different treatments. As shown in Fig. 3A, single treatment with either DOX or PIP showed a comparable 2-fold significant reduction in PI3K protein levels compared to untreated cells, whereas combining PIP to DOX resulted in an additional 2-fold reduction relative to DOX treatment alone. To confirm suppression of downstream targets, p-Akt (Fig. 3B) and mTOR (Fig. 3C) levels were estimated in cell lysates. In line with PI3K findings, DOX treatment resulted in significantly lower levels of p-Akt and mTOR showing a 20 and 29% reduction, respectively. Adding PIP to DOX consistently enhanced curbing p-Akt and mTOR protein levels showing a 29 and 60% significant reduction, respectively, compared to single treatment with DOX. These alterations were mirrored by an opposing pattern in PTEN expression whose levels were elevated by two folds in response to DOX and PIP single treatments while combined treatment caused the most profound 4-fold surge (Fig. 3D).

### Combining PIP to DOX reduces tumor size and improves survival of tumor-bearing mice

**Figure 4:**
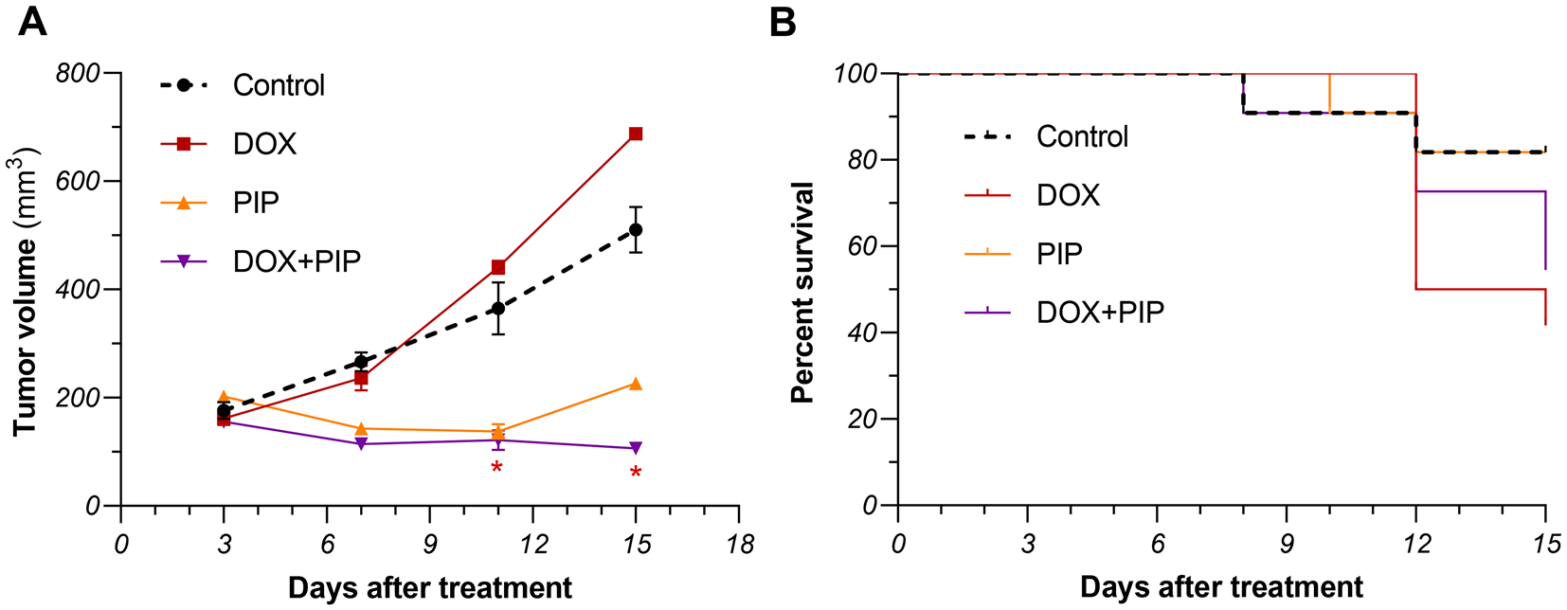
Effect of combining PIP to DOX on tumor volume and survival of EAC-tumor bearing mice. (**A**) Tumor volume (mm^3^) estimated at days 3, 7, 11, and 15 post treatment. (**B**) Survival curve in mice treated with DOX and PIP, alone and combined compared to control. Survival analysis was performed using Kaplan Meier test. Tumor volume data are square root-transformed and were analyzed using one-way ANOVA followed by Tukey’s multiple comparison test and significance was inferred for *P* < 0.05. Results are presented as means ± S.D. *Significant *vs* DOX-treated group. PIP, piperine; DOX, doxorubicin.

To explore the impact of combining PIP to DOX treatment in vivo, EAC solid tumor model was used in mice and tumor volumes were estimated at different time points. As shown in Fig. 4A, combining PIP to DOX resulted in significantly lower tumor size compared to DOX alone reaching 72% (*P* = 0.024) and 84.5% (*P* = 0.0008), at days 11 and 15 post treatment, respectively. Moreover, combining PIP to DOX improved survival rates in mice compared to their DOX-treated counterparts (Fig. 4B).

### PIP protects against DOX-induced cardiotoxicity

**Figure 5:**
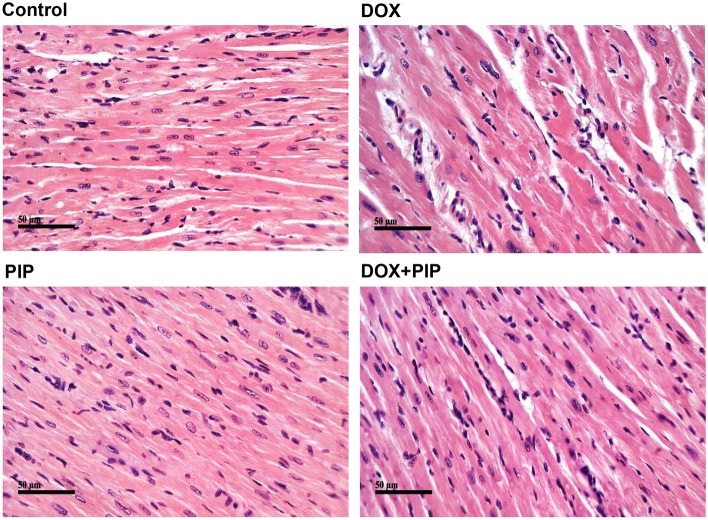
Effect of combining PIP to DOX on histopathological alterations in cardiac tissue sections of EAC-tumor bearing mice. Representative photomicrographs of H&E-stained cardiac tissue sections (x100) in different treatment groups.

As shown in Fig. 5, H&E-stained cardiac sections from the DOX-treated group showed focal areas of widely separated cardiomyocytes with intercellular inflammatory cells. Additionally, many cardiac cells demonstrated fragmentation of myofibrils with or without pyknotic nuclei. PIP-treated group showed apparently normal cardiomyocytes without abnormal tissue alteration. On the other hand, DOX + PIP group showed more intact cardiac muscles cells alternated with lesser numbers of degenerated cells with pyknotic nuclei. Milder inflammatory cells infiltration was observed suggesting a protective role for PIP against DOX-induced cardiotoxicity.

### PIP enhances DOX-induced amelioration of histopathological signs of malignancy and necrosis in tumor sections

**Figure 6:**
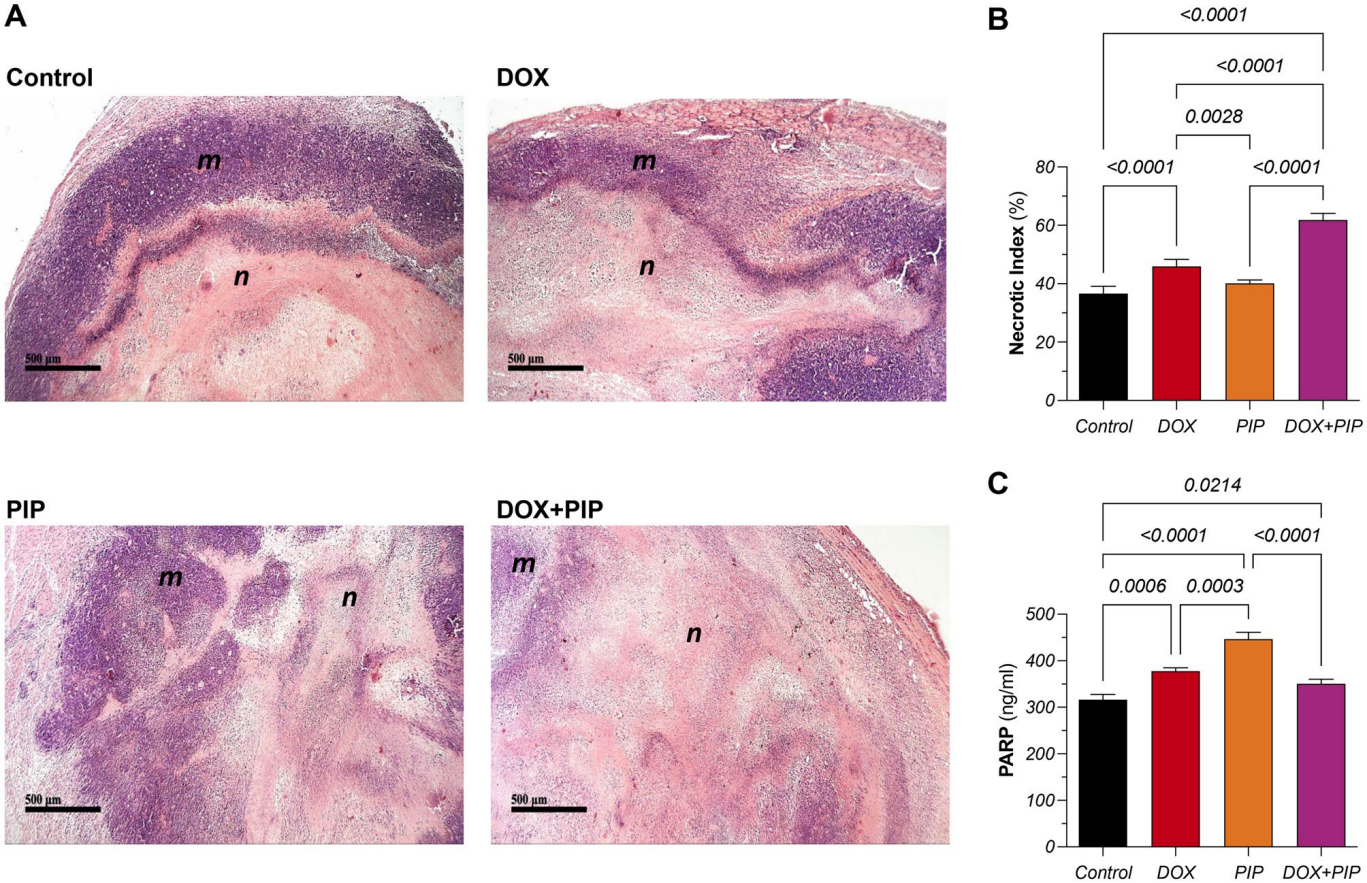
Effect of combining PIP to DOX on tumor histopathology, necrotic indices, and protein levels of cleaved PARP in EAC-tumor bearing mice. (**A**) Representative photomicrographs of H&E-stained tumor sections (x100) in control, DOX-, PIP-, and DOX + PIP-treated groups and (**B**) their corresponding necrotic indices. (**C**) Protein levels of cleaved PARP in tumor tissues of different groups estimated using ELISA. Statistical difference was tested using one-way ANOVA followed by Tukey’s multiple comparison test and significance was inferred for *P* < 0.05. Results are presented as means ± S.D. *n* indicates *necrosis* and* m* indicates *malignant cells*. PIP, piperine; DOX, doxorubicin; PARP, poly-ADP ribose polymerase.

Histopathological examination of control tumor samples showed pleiomorphic malignant cells with mitotic appearance (Fig. 6A). On the other hand, DOX-treated group showed multiple foci of viable tumor cells surrounded by necrotic tumor cells with pyknotic nuclei. Tumor sections of the DOX + PIP group showed large areas of necrosis and displayed the highest necrotic index, almost 2-fold higher than that observed in the DOX group (Fig. 6B).

### PIP enhances apoptosis via PARP cleavage in EAC-tumor bearing mice

To further explore how exactly PIP enhances DOX tumor killing capacity, cleaved PARP was estimated in tumor tissue homogenates. As shown in Fig. 6C, DOX treatment resulted in a marginal, yet significant 19% increase in cleaved PARP protein levels compared to control while combining PIP to DOX resulted in no significant difference in its levels. However, the highest significant increase was observed in the PIP treated group reaching 41% compared to the untreated control group.

### Combining PIP to DOX modulates ALDH-1 levels in EAC-tumor bearing mice

**Figure 7:**
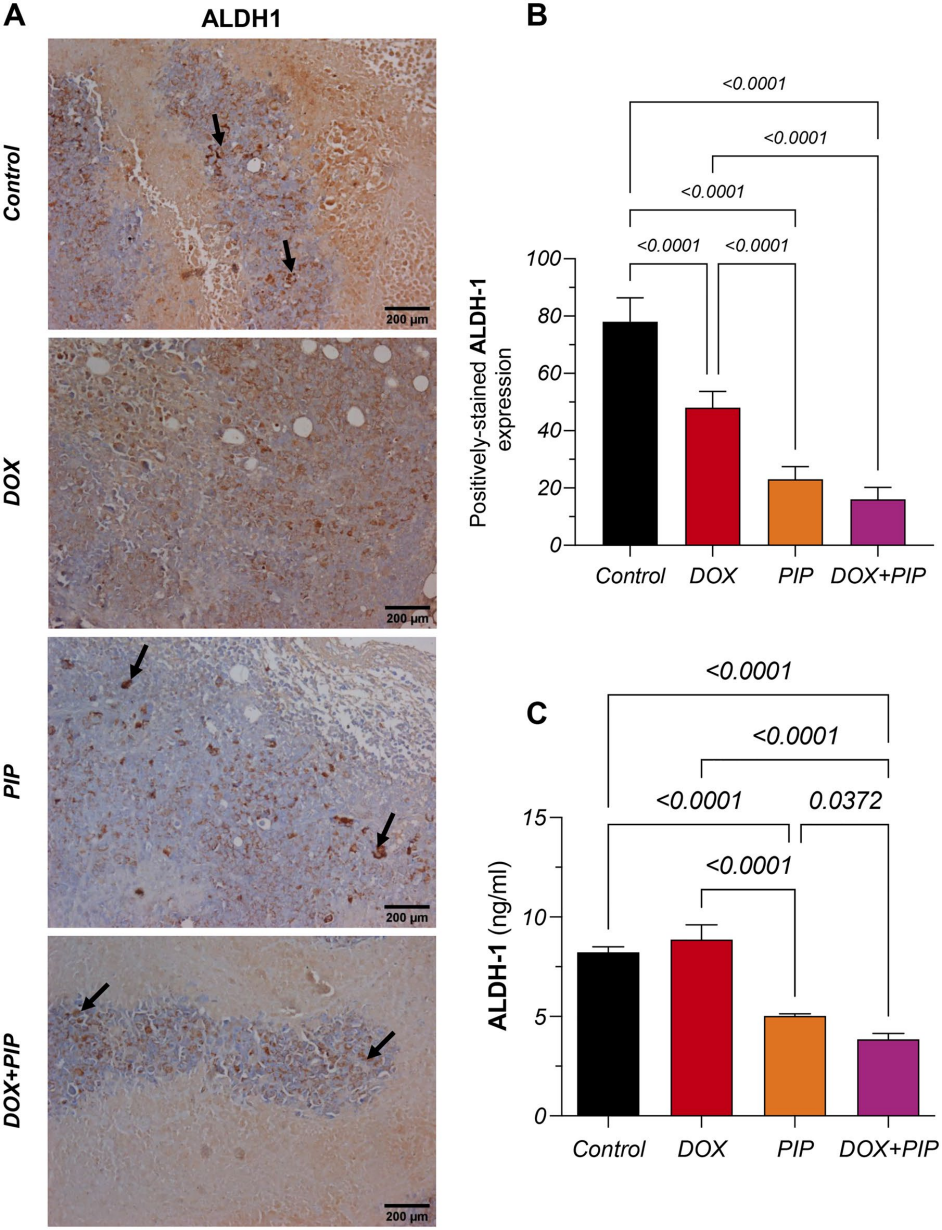
Effect of combining PIP to DOX on levels of ALDH-1 in EAC-tumor bearing mice. (**A**) Representative photomicrographs (DAB, x200) of tumor sections from control, DOX-, PIP-, and DOX + PIP-treated EAC-tumor bearing mice for ALDH-1 immunohistochmeical expression. (**B**) Semi-quantitative analysis for ALDH-1-stained sections. (**C**) Protein levels of ALDH-1 estimated by ELISA. Statistical difference was tested using one-way ANOVA followed by Tukey’s multiple comparison test and significance was inferred for *P* < 0.05. Results are presented as means ± S.D. PIP, piperine; DOX, doxorubicin; ALDH-1, aldehyde dehydrogenase-1.

To validate the alterations in ALDH-1 levels observed in vitro in response to DOX, PIP, and their combination, immunoreactivity against ALDH-1 was estimated using immunohistochemistry (Fig. 7A) in tumor tissue sections and subsequently confirmed using ELISA in tumor tissue homogenates. As shown in Fig. 7B,C, DOX treatment resulted in a 38% reduction in ALDH-1 expression levels as estimated by immunohistochemistry, however, no significant changes were observed in its level in the corresponding tumor tissue homogenates when estimated using ELISA. PIP treatment, on the other hand, significantly reduced ALDH-1 expression by 71% and 39% compared to the control in EAC tumor-bearing mice, when measured by immunohistochemistry and ELISA, respectively. Interestingly, when PIP was combined with DOX, an additional suppression was observed resulting in a 56% reduction in ALDH-1 immunohistochemical expression and a 67% reduction in its levels in tumor homogenates, relative to DOX treatment alone.

Furthermore, treatment with PIP resulted in 71% and 39% reductions in ALDH-1 levels compared to control EAC tumor-bearing mice, as estimated by immunohistochemistry and ELISA, respectively. Combining PIP to DOX, however, caused further reductions in ALDH-1 levels reaching 56% and 67% relative to DOX, as evidenced by immunohistochemistry and ELISA methods, respectively. These results might allude to the capacity of PIP to modulate the impact DOX has on CSCs.

### Combining PIP to DOX modulates PTEN/PI3K/Akt/mTOR pathway in EAC tumor-bearing mice

**Figure 8:**
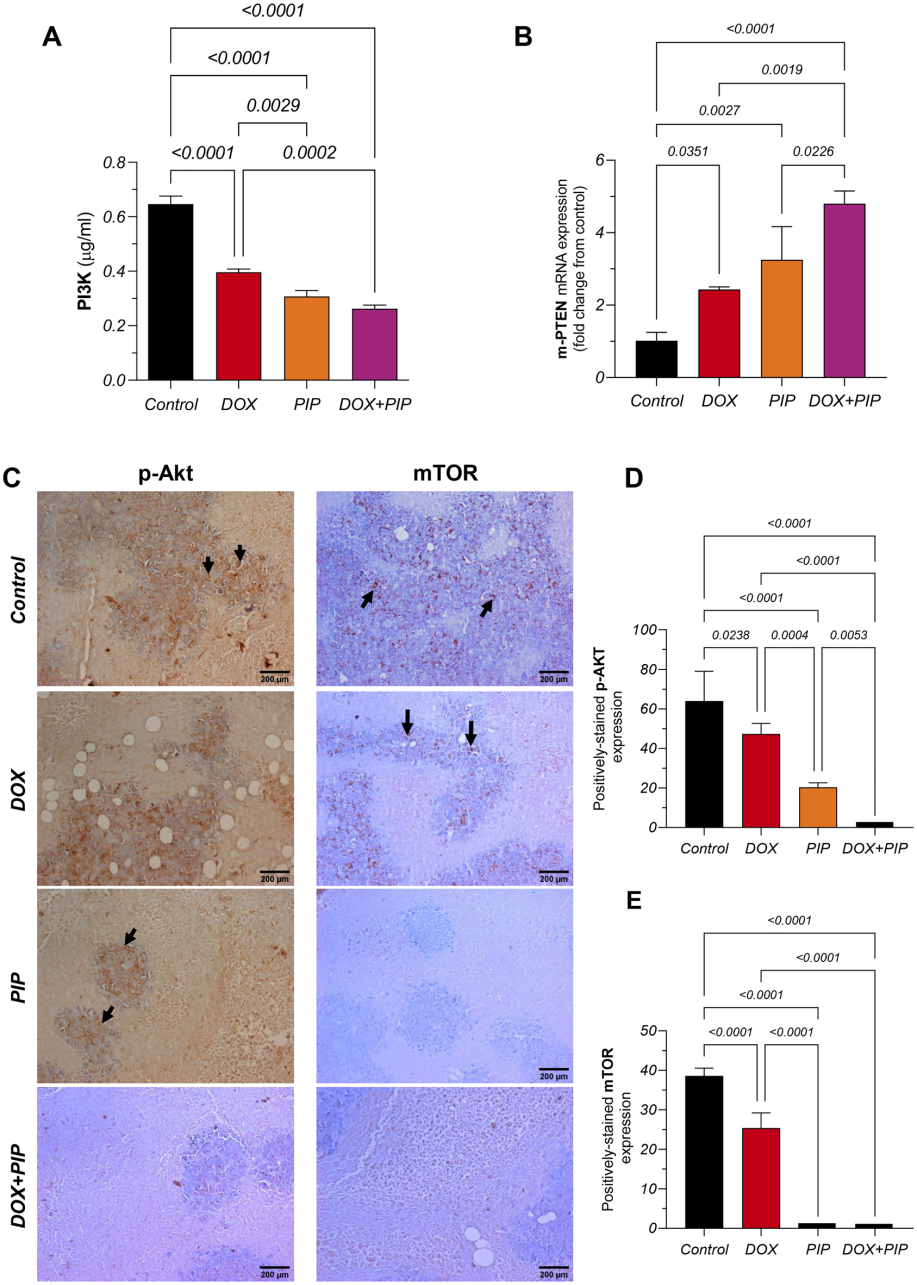
Effect of combining PIP to DOX on levels of PI3K, p-Akt, mTOR, and PTEN in EAC-tumor bearing mice. (**A**) Protein levels of PI3K; (**B**) PTEN gene expression; (**C**) Representative photomicrographs (DAB, x200) of tumor sections from control, DOX-, PIP-, and DOX + PIP-treated EAC-tumor bearing mice for p-Akt and mTOR immunohistochemical expression. Semi-quantitative analyses for stained sections representing the percentage of positive expression of (**D**) p-Akt and (**E**) mTOR in different groups. Protein levels in **(A)** were estimated using ELISA. Relative gene expression in (**B**) is expressed as fold change from control and was determined by qRT-PCR technique using delta-delta Ct (ΔΔCt) following normalization to the housekeeping β-actin gene. Statistical difference was tested using one-way ANOVA followed by Tukey’s multiple comparison test and significance was inferred for *P* < 0.05. Results are presented as means ± S.D. Brownish cytoplasmic discoloration represents positive expression as indicated by black arrows in (**C**).

To gain further mechanistic insights into the potential underpinnings contributing to PIP's impact on DOX efficacy and chemoresistance and to further substantiate the in vitro results, key signaling molecules within the PI3K/Akt/mTOR pathway, well-characterized for their tumorigenic role in TNBC, were investigated. As shown in Fig. 8A, DOX treatment resulted in a significant 39% reduction in PI3K levels in tumor homogenates compared to control. Combining PIP to DOX resulted in a further reduction reaching 34% compared to DOX alone. The ensuing role of PI3K in phosphorylating Akt is opposed by PTEN, the expression of which was quantified to gauge the impact of treatments on modulating this pathway activity. DOX, PIP, and their combination elicited 2.4-, 3.3-, and 4.8-fold upregulation in PTEN expression, as compared to control untreated mice (Fig. 8B). Immunohistochemical assessments provided further insights (Fig. 8C) showing reductions in p-Akt levels upon DOX and PIP treatments reaching 47.4% and 20.4%, respectively, with undetectable expression in the DOX + PIP group, as compared to control (Fig. 8D). In parallel, phosphorylated mTOR protein expression was markedly inhibited with DOX treatment reaching 25.4% compared to control, whereas both PIP and the combined treatment completely abrogated mTOR expression (Fig. 8E). Collectively, these findings underscore the likely inhibitory effects of PIP, individually and in combination with DOX, on key mediators of the PI3K/AKT/mTOR signaling cascade, highlighting the potential combinatorial benefit in enhancing the chemotherapeutic response to DOX and curbing its resistance.

## Discussion

Chemotherapy remains the standard-of-care to many TNBC patients who do not benefit from endocrine and HER2 targeted therapeutic modalities. Relying solely on chemotherapy entails vulnerability towards the well-known side effects typically presenting with systemic therapy as well as regimen-specific adverse events. Additionally, chemoresistance may be encountered leading to therapeutic failure and tumor recurrence, thus representing another barrier to complete remission^6^. Although a well-characterized mechanism explaining chemoresistance in TNBC is lacking owing to its heterogeneous nature, several molecular processes have been implicated. The PI3K signaling pathway, in particular, has drawn much attention in TNBC mainly due to the observation that many TNBC patients frequently present with mutations in this pathway or its negative regulator, PTEN. In fact, frequent aberrations in PI3K/Akt/mTOR only come second to that of TP53 in TNBC^9^. Beyond its well-documented contribution to cancer sustenance, PI3K signaling entangled association with chemoresistance mechanisms including drug-efflux proteins, cancer cell stemness, and regulation of PARP activity^21^, makes it a compelling target in TNBC which was underscored in numerous clinical trials^9,22^.

Piperine (herein referred to as PIP), the principal active constituent of black pepper, has depicted a broad spectrum of anticancer properties across various malignancies, showcasing its antiproliferative, pro-apoptotic, and antimetastatic capabilities. Its efficacy is not confined to a single type of cancer, rather, PIP has been effective in an array of cancers including colorectal^12^, prostate^23^, gastric^24^, and cervical^25^ cancers. Remarkably, its impact extends to more challenging and resistant cancer types, such as glioblastoma multiforme^26^ and TNBC^27^. PIP has garnered recognition not only for its inherent anticancer properties but also for its significant role in counteracting chemoresistance, thereby enhancing the efficacy of conventional chemotherapy. It achieves this by modulating several critical molecular pathways, notably including the PI3K/Akt/mTOR^28^. Furthermore, PIP’s ability to inhibit EMT and hence the pool of CSCs marks its significant role in alleviating chemoresistance^29^. Therefore, the current study aimed at probing the capacity of PIP to sensitize TNBC cells towards DOX, a cornerstone in TNBC regimens limited by either innate or acquired chemoresistance. Additionally, it explores whether the previously reported pleiotropic effects of PIP could be leveraged towards diminishing the prototypical cardiotoxicity induced by DOX treatment.

To achieve the aim of the current investigation, cytotoxicity studies were conducted on the TNBC cells, MDA-MB-231, treated with DOX, PIP, and their combination. Our results showed that combining PIP to DOX enhanced the cytotoxic effect of DOX by eliciting a synergistic interaction. Our current study finding is corroborated by previous reports alluding to the chemosensitizing capacity of PIP to other chemotherapeutic agents in estrogen-dependent^30,31^ and HER2-positive^32^ breast cancer subtypes. The cytotoxic effect of PIP as a single treatment on TNBC cell lines was also previously reported^27^.

To better understand the likely mechanisms contributing to PIP-induced enhancement of DOX cytotoxicity and founded on the previously reported implications of PI3K signaling in TNBC, we explored the impact of PIP on PI3K/Akt/mTOR signaling cascade along with its affiliated key players in chemoresistance. In the current investigation, DOX alone resulted in a significant suppression of the PI3K signaling, and combining PIP to DOX was interestingly found to strengthen this inhibitory effect which was further confirmed by the opposing expression pattern observed in PTEN levels both in vitro in MDA-MB-231 cells and in EAC-tumor bearing mice in vivo. The measurement of PTEN was strategically conducted as an indicator to validate the inhibition of the PI3K/Akt/mTOR pathway, given PTEN’s role as a critical negative regulator of this signaling axis. The increase in PTEN expression, therefore, serves as a corroborative marker of this pathway suppression^33^. The augmented suppression depicted both in vitro and in vivo might explicate the PIP-induced enhancement of TNBC sensitivity to DOX in these settings. It is worth mentioning that the current study is the first to suggest the capacity of PIP to modulate the PI3K/Akt/mTOR signaling in TNBC, only preceded by a previous report of p-Akt suppression exerted by PIP as a single treatment^27^. Furthermore, we report for the first time that the chemosensitizing effect of PIP to DOX might be mediated by the suppression of PI3K/Akt/mTOR signaling and the induction of PTEN expression.

Another plausible rationale is the capacity of PIP to engage with complementary pathways associated with PI3K/Akt/mTOR signaling network mediating its role in chemoresistance such as those relating to CSCs^34,35^ or response to DNA damage^36,37^ typically induced by DOX treatment^10,11,38^. To test this hypothesis, the CSC surrogate marker, ALDH-1, as well as the pro-apoptotic enzyme, PARP-1, were estimated. Beyond serving as a surrogate marker of CSCs in cancer at large^39^ or specifically in TNBC^40,41^, the estimation of ALDH-1 levels is also suggested as a prognostic tool in TNBC patients^42,43^. Increased ALDH-1 expression is associated with tumor cell aggressiveness and has been linked to increased activity in PI3K/Akt/mTOR signaling in breast cancer^44,45^. In the current study, we quantified ALDH-1 using ELISA for both the in vitro and in vivo arms, while immunohistochemistry was reserved for in vivo experiments. That being the case, we identified discrepancies in ALDH-1 levels with DOX treatment, which significantly reduced its levels in vitro as per the ELISA results as well as its immunohistochemical expression in vivo. This reduction, however, was not mirrored in vivo when measured by ELISA, where the levels in DOX-treated tissue homogenates were insignificantly altered. The discrepancy in ALDH-1 levels could be explained in light of the apparent differences between the two experimental settings in which the restrictive/controlled conditions of single-cell in vitro cultures do not recapitulate complexities of the tumor microenvironment in vivo where a myriad of tumor cell sub-populations, as well as immune cells, might impact the response to therapy. Furthermore, this discrepancy underscores how different assessment methods can impact the interpretation of changes in expression levels. For instance, the variation between ELISA and immunohistochemistry observed in the in vivo arm is likely rooted in the inherent differences in sensitivity and specificity between these techniques. ELISA, known for its quantitative precision and higher sensitivity, excels in detecting and quantifying markers in homogenized samples. Conversely, immunohistochemistry provides spatial context but may have lower sensitivity. Importantly, the immunohistochemistry results in our study, which exclusively reported cytoplasmic expression, might more closely represent the true status of ALDH-1, being a cytoplasmic marker for CSCs, as this specificity is unattainable in whole tissue homogenates analyzed by ELISA. Therefore, the immunohistochemistry context and localization may offer a more accurate portrayal of ALDH-1 distribution within tumor tissues. On the other hand, we found that single treatment with PIP resulted in a significant suppression in ALDH-1 levels both in vitro and in vivo*,* and combining PIP to DOX achieved the lowest protein levels in cell lysates and tumor tissue homogenates as well as its immunoreactivity in tissue sections. This marked reduction in ALDH-1 in the combination group paralleled the most profound impact on tumor shrinkage and necrotic index in DOX + PIP-treated mice. These results, despite novelty, align with prior research demonstrating the capacity of PIP to intervene with CSCs in other breast cancer cell lines decreasing ALDH-1 levels and compromising mammosphere formation in hormone responsive MCF-7 and mesenchymal TNBC SUM 159 cells^46^. The heightened necrotic indices observed in the combination group was yet another motive for us to estimate the pro-apoptotic enzyme, PARP, other than its association with the PI3K signaling, as previously mentioned. Single treatment with PIP resulted in the highest levels of cleaved PARP, however, combining PIP to DOX did not result in any significant difference compared to DOX single treatment. These results suggest that the augmented antiproliferative capacity of DOX when combined with PIP might be mediated by a necrotic rather than an apoptotic mechanism. It is worth mentioning that PIP alone caused the highest upsurge in PARP level, aligning with previous studies^30,47^ and suggesting a different mode of cytotoxicity when used alone.

Another intriguing finding of the current investigation is the reduction observed in DOX-induced cardiotoxicity which might partly contribute to the improved survival observed in the combination group. This finding is supported by two recent studies where the protective effect of PIP against DOX-induced cardiotoxicity was reported in mice receiving a single high dose of DOX^48^ as well as with chronic use of DOX in mammary tumor-bearing rats^49^. Although in the latter study, the cardioprotective effects were rather attributed to the whole *P. nigrum* extract instead of a specific compound.

The current study, however, holds several limitations. First, in vitro assessment of the contribution of PIP on PI3K/Akt/mTOR-mediated DOX chemoresistance could have been more befittingly investigated in DOX-resistant cell line to confirm the reported findings. Another limitation lies in the fact that our study was conducted using a single TNBC cell line. Considering the heterogeneity of TNBC, future research involving multiple cell lines would be beneficial to validate and extrapolate the presented findings to other TNBC subtypes. Additionally, inferences from the current in vivo investigations are restrained by the adopted non-specific model of breast carcinoma, and therefore future investigations using a more specific xenograft animal model or the mammary intraductal (MIND) engraftment of the 4T1 carcinoma cells are highly recommended. Another limitation is related to the reliance on ALDH-1 as a marker for CSCs in our study. The use of additional CSC markers, such as CD24 and CD44, could have provided a more comprehensive evaluation of the CSC population. This could be achieved by flow cytometric analysis to phenotypically characterize the CD44+/CD24− subpopulation that is peculiar to breast CSCs. We also believe that future studies employing a specific inhibitor for the PI3K/AKT/mTOR pathway and its key molecular targets, such as LY294002 or rapamycin, would further validate the implication of this pathway in TNBC chemoresistance and how DOX and PIP combination can effectively disrupt it.

**Figure 9:**
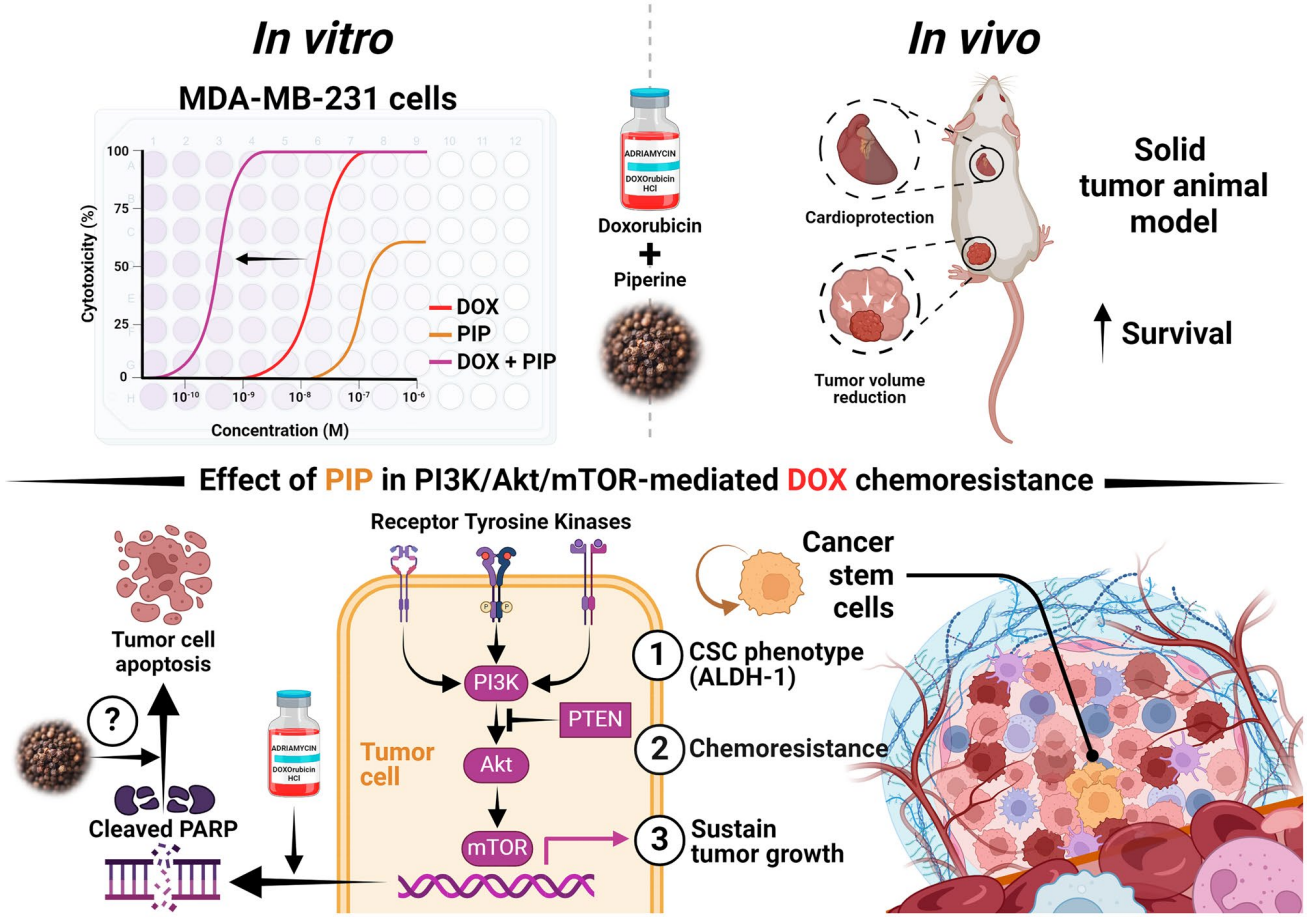
Schematic diagram depicting the proposed mechanism by which PIP combats DOX chemoresistance in TNBC. PIP, in combination with DOX, exerts a dual effect by suppressing the PI3K/Akt/mTOR pathway, crucial for tumor cell survival and subsequently reducing ALDH-1, a surrogate marker for CSCs. This concerted action may mitigate chemoresistance by diminishing the population of CSCs responsible for mediating resistance to DOX chemotherapy. This hypothesis was tested in vitro using MDA-MB-231 cells and in vivo using an animal model of EAC solid tumor. ALDH-1, aldehyde dehydrogenase-1; CSCs, cancer stem cells, DOX, doxorubicin; EAC, Ehrlich ascites carcinoma; PIP, piperine; TNBC: triple-negative breast cancer.

Taken together, the present study demonstrated the capacity of PIP to enhance the cytotoxic effect of DOX exhibiting a synergistic interaction in TNBC cells. The proposed underlying mechanism is the interference with the PI3K/Akt/mTOR signaling with a likely impact on CSCs and a subsequent enhancement of necrosis rather than apoptosis. The present study also incites further clinical investigations in which PIP might serve as a potential adjuvant to enhance the efficacy and safety profiles of DOX in breast cancer patients particularly the ones with the triple-negative phenotype. A schematic diagram summarizing the proposed mechanistic insights of the current study is provided in Fig. 9.

## Material and methods

### Piperine isolation and purification

Black pepper (*P. nigrum*) whole fruits commonly used as food spice were obtained from a local herbal store in Cairo, Egypt whereas the collection of the fruits complies with the IUCN Policy statement on Research Involving Species at Risk of Extinction and the Convention on Trade in Endangered Species of Wild Fauna and Flora. Identity of plant material was kindly verified by Dr. Mohamed Al-Gebaly, Professor of Plant Taxonomy at Ain Shams University, and a voucher specimen designated **PN-F01** was deposited in the repository of the Faculty of Pharmacy at The British University in Egypt. Piperine (PIP) was isolated and purified from the ethanolic extracts of *P. nigrum* fruits using reflux and recrystallization procedures, as previously described^50^. Briefly, ethanolic extraction was carried out for 250 gm of crushed *P. nigrum* fruits using Soxhlet apparatus (Glassco Laboratory Equipment Pvt. Ltd., India). The obtained ethanolic extract was then concentrated using Büchi® Rotavapor (BÜCHI Labortechnik AG, Switzerland) to yield 50 gm of crude pepper extract. Impurities from the crude extract were removed by adding 1 gm of KOH (Sigma-Aldrich, Germany) and PIP was precipitated by adding an excess of water. Vacuum filtration was carried out using Buchner apparatus (Glassco Laboratory Equipment Pvt. Ltd., India). Recrystallization of PIP was then carried out by adding an excess of ethanol to obtain a yield of 1.8 gm.

### Identification of isolated piperine using 1D-NMR (^1^H and ^13^C)

The structural identity of the PIP yield isolated from the *P. nigrum* ethanolic extract was verified using one-dimensional ^1^H and ^13^C nuclear magnetic resonance spectroscopy (1D-^1^H and ^13^C NMR). The 1D-NMR data were obtained in CDCl_4_ on a Bruker AVANCE spectrometer, observing ^1^H and ^13^C (400 and 100 MHz, respectively). The chemical shifts (δ) were measured in ppm, related to TMS signal at 0.00 ppm as an internal reference.

### Purity of PIP via HPLC–PDA

Isolated PIP was prepared in a concentration of 10 μg/mL in methanol to confirm its purity using high performance liquid chromatography coupled to photodiode array detector (HPLC–PDA). Authenticity of PIP was validated using Thermo Fisher UPLC Ultimate 3000 Complete Ultra Performance Liquid Chromatography (USA) coupled to UV Detector (PDA 3000 RS, USA) and an autosampler (WPS-3000TRS, Thermo scientific USA). HPLC column was used (Kromasil C18, 250 × 4.6, particle size 5µ). The HPLC method of analysis was adopted from a previous study with minor modifications^51^. The isocratic elution of PIP was carried out for 30 min using 0.1% orthophosphoric acid in water (solvent A) and acetonitrile (solvent B). The injection volume was 10 µL with a flow rate of 1 mL/min and ambient column temperature. PIP was measured at a wavelength of 345 nm, as previously described^52^.

### In vitro studies

#### Cell culture

An in vitro study was carried out using the TNBC cell line, MDA-MB-231, to assess the chemo-sensitizing potential of PIP on DOX treatment. Cells were obtained from the American Type Culture Collection (ATCC, USA) and cultured in T75 flasks (Greiner Bio-One, Germany) using complete Dulbecco’s Modified Eagle’s Medium (DMEM; Gibco, USA) supplemented with 10% fetal bovine serum (FBS; Gibco USA), and 1% antimycotic antibiotic mixture (Gibco, USA). Cells were maintained in a CO_2_ incubator at 37 °C (Thermo Fischer Scientific, USA) and serially split at 70–80% confluency.

#### Cytotoxicity and synergy studies

Cytotoxicity assays were carried out and half-maximal inhibitory concentrations (IC_50_) of different drug treatments were estimated using 3-(4,5-dimethylthiazol-2-yl)-2,5-diphenyltetrazolium bromide (MTT) (SERVA, Germany). Briefly, cells were seeded in 96 well plates at a density of 15 × 10^3^ cells per well and allowed to incubate for 24 h. Cells were then treated with a ten-fold serial dilution (0.02–200 µM) of DOX (Pfizer, USA). Cells were also treated with PIP, alone and combined with DOX. PIP’s concentrations used in the combination were 100 and 200 µM. The selection of these two concentrations was based on pilot studies that aimed to determine the optimal concentration range of PIP that can achieve synergy with DOX. Cells were incubated with treatments for 72 h. Culture media was then discarded, and 5 mg/mL of MTT was added to each well and incubated for 2 h. Culture plates were then decanted and 200 µL of DMSO was added in each well to dissolve and visualize the purple formazan crystals. Absorbance was then measured using a microplate reader (BioTek, USA) at 570 nm wavelength. Viability was calculated as a percentage of absorbance detected in treated cells subtracted from that of control. The IC_50_ values of different drug treatments were calculated using GraphPad Prism V9.0 software (GraphPad Inc., USA). The combination index (CI) was then calculated according to the following formula to study the nature of interaction between treatments^53^:

$${\text{CI}} = \left[ {{\text{DOX}}\;{\text{IC}}_{50} \;{\text{in}}\;{\text{combination}}/{\text{DOX}}\;{\text{IC}}_{50} \;{\text{alone}}} \right] + [{\text{PIP}}\;{\text{conc}}.\;{\text{in}}\;{\text{combination}}/{\text{PIP}}\;{\text{IC}}_{50} \;{\text{alone}}]$$

The interaction is described as additive if CI = 1, synergistic if CI < 1, and antagonistic if CI > 1.

#### Estimation of PTEN gene expression levels in TNBC cells

Total RNA was isolated from cell lysates using the Direct-zol RNA Miniprep Plus kit (Cat# R2072, ZYMO RESEARCH CORP., USA). The RNA’s quantity and quality were then evaluated based on the A260/A280 absorbance ratio. Subsequently, reverse transcription of the extracted RNA and PCR steps were conducted simultaneously using the SuperScript IV One-Step RT-PCR kit (Cat# 12594100, Thermo Fisher Scientific, Waltham, MA, USA). The data were represented as Cycle threshold (Ct) values. The relative expression levels were calculated using the delta-delta Ct (ΔΔCt) method, with β-actin serving as the internal control gene^54^. The primer sequence of the human PTEN (h-PEN; NCBI accession no.: NM_000314.8) gene used is as follows: forward 5′-TGAGTTCCCTCAGCCGTTACCT-3′ and reverse 5′-GAGGTTTCCTCTGGTCCTGGTA-3′. The primer sequence of the human β-actin (NCBI accession no.: NM_001101.5) gene used is: forward 5′-CACCATTGGCAATGAGCGGTTC-3′ and reverse 5′-AGGTCTTTGCGGATGTCCACGT-3′.

#### Estimation of ALDH-1, PI3K, p-Akt, and mTOR protein levels in TNBC cells

Cells were cultured in 6-well plates, incubated overnight and then treated with DOX and PIP, alone and combined, for 72 h. Cells were then harvested and the following parameters were estimated in cell lysates using ELISA adhering to the manufacturers’ instructions: aldehyde dehydrogenase-1 (ALDH-1; Cloud-Clone Corp., USA; CAS# SEE824Hu); phosphatidylinositol-3-kinase (PI3K; MyBioSource, USA; CAT# MBS268899); phosphorylated Akt (p-Akt; MyBioSource, USA; CAT#: MBS167855); mammalian target of rapamycin (mTOR; MyBioSource, USA; CAT# MBS2505637).

### In vivo studies

#### Animals

Female Swiss albino mice, 6 weeks of age and weighing 20–25 g, were used in the current study. Animals were allowed to acclimatize two weeks prior to model induction. Throughout the experimental protocol, mice were kept under constant conditions of temperature, humidity, and light/dark cycles. Ethical approval for the current study protocol was sought and granted by the Ethics Committee at the Faculty of Pharmacy, the British University in Egypt (EX-1907). All measures were taken to reduce animal pain and suffering during model induction and other experimental procedures in accordance with the Animal Research: Reporting of In Vivo Experiments (ARRIVE) guidelines. All methods were performed in accordance with the relevant guidelines and regulations.

#### Experimental model and design

Ehrlich ascites carcinoma (EAC) solid tumor model was used in the current study. EAC cells were first grown in the peritoneum of a female Swiss albino mouse for 8 days and the tumor cell suspension was subsequently harvested from the ascitic fluid. EAC cells were then inoculated intramuscularly into the left thighs of mice belonging to different groups at a density of ~ 2 × 10^6^ cells/mouse suspended in 0.2 mL saline for each. Selection of animals, treatment groups, and regimens was made based on our prior work alongside work form other groups and was initiated 5 days after inoculation (henceforth referred to as day 0). Mice were then randomly distributed to the following 4 groups (n = 10): (I) *Control:* Mice only received drug vehicles; (II) *DOX:* Mice received a single weekly dose of DOX at 4 mg/kg, administered intraperitoneally^55,56^; (III) *PIP:* Mice received a daily dose of PIP at 50 mg/kg, administered intraperitoneally^57^; (IV) *DOX + PIP:* Mice received a combination of the aforementioned treatments at their respective regimens. Another normal untreated group was used for heart tissue collection at the end of the experiment. For the selection of doses for the combination-treated group, we adopted a common approach based on the use of the same doses of each drug in the combination therapy as those used in individual treatments, aiming to achieve a significant enhancement in efficacy. Mice were sacrificed after 15 days of treatment. Carbon dioxide inhalation was used for euthanasia. Heart and tumor tissues were then carefully excised and either preserved in 10% formalin for histopathological evaluation and immunohistochemical investigations or snap-frozen and stored at − 80 °C for subsequent biochemical analyses.

#### Tumor size estimation

Tumor dimensions were measured at days 3, 7, 11, and 15 post-treatment using a digital caliper. Tumor size was then calculated using the following equation^58^:

$${\text{Tumor}}\;{\text{size}} = {\text{Length}}\;\left( {{\text{mm}}} \right) \times {\text{Width}}^{2} \;\left( {{\text{mm}}^{2} } \right) \times 0.52$$

#### Histopathological evaluation of tumors and heart tissues

Tumor and heart tissues fixed in 10% formalin were pruned, dehydrated in serial ascending grades of ethanol, washed in xylene, and then embedded in paraffin. Preserved paraffin-embedded tissues were then cut into 4 μm thick sections using a rotary microtome (microTEC, Duisburg, Germany) and stained using hematoxylin and eosin (H&E). Ten random fields per section per mouse were photographed at a magnification power of ×100 using a light microscope (Zeiss, Oberkochen, Germany). Images of tumor tissue sections were used to estimate the necrotic index (NI) using Leica application computer analyzer system (Leica Microsystems, Switzerland).

#### Immunohistochemical estimation of ALDH-1, p-Akt, and mTOR in tumor tissue sections

Immunohistochemical detection of diverse tumorigenic parameters was performed in the current study adopting the avidin–biotin peroxidase complex method^59^. We utilized primary monoclonal antibodies specific to ALDH-1A1 (Santa Cruz Biotechnology, Inc., Santa Cruz, USA, CAT#: sc-166362), p-Akt Ser473 (Santa Cruz Biotechnology, Inc., Santa Cruz, USA, CAT#: sc-514032), and p-mTOR Ser2481 (Santa Cruz Biotechnology, Inc., Santa Cruz, USA, CAT#: sc-293089) at a dilution ratio of 1:100 for estimation in tumor tissue sections. Color development was facilitated using a preformed streptavidin–biotin complex with peroxidase and diaminobenzidine, adhering to the manufacturer’s protocol (Dako, Denmark). Following the immunoreactivity, sections were counterstained with Mayer’s hematoxylin. The semi-quantitative expression of ALDH-1, p-Akt, and mTOR was evaluated in ten randomly selected fields per section for each slide.

#### Estimation of PTEN gene expression levels in tumor tissues

RNA extraction, reverse transcription, and PCR steps were performed on tumor sections following the manufacturers’ instructions listed in “Estimation of PTEN gene expression levels in TNBC cells” section. The primer sequence of the mouse PTEN (m-PEN; NCBI accession no.: NM_008960.2) gene used is as follows: forward 5′-TGAGTTCCCTCAGCCATTGCCT-3′ and reverse 5′-GAGGTTTCCTCTGGTCCTGGTA-3′. The primer sequence of the mouse β-actin (NCBI accession no.: NM_007393.5) gene used is: forward 5′-CATTGCTGACAGGATGCAGAAGG-3′ and reverse 5′-TGCTGGAAGGTGGACAGTGAGG-3′.

#### Estimation of ALDH-1, cleaved PARP, and PI3K protein levels in tumor tissues

Tumor tissues were homogenized in ice-cold phosphate buffered saline (PBS, Lonza, Switzerland) as 10% weight/volume using a tissue homogenizer. The following parameters were then measured in tissue homogenates using ELISA adhering to the manufacturers’ instructions: ALDH-1 (Cloud-Clone Corp., USA; CAT# SEE824Hu); cleaved PARP (LSBio Inc., USA; CAT# LS-F15453), and PI3K (MyBioSource, USA; CAT# MBS268899).

### Statistical analysis

Data are expressed as mean ± standard deviation. Data were tested for statistical difference and visualized using GraphPad Prism software V9.0 (GraphPad, Inc., USA). Survival analysis was performed using Kaplan–Meier test, while other parameters were tested using one-way ANOVA followed by Tukey's multiple comparisons test. Tumor volume data were square root-transformed following Brown–Forsythe test. Significant differences were inferred for *P* values below a threshold value of 0.05.

## Data Availability

All data presented and analyzed in the current study is available upon reasonable request from the corresponding author.
